# An Engineered Arginase FC Protein Inhibits Tumor Growth *In Vitro* and *In Vivo*

**DOI:** 10.1155/2013/423129

**Published:** 2013-05-08

**Authors:** Lihua Li, Yan Wang, Jun Chen, Bi Cheng, Jiehua Hu, Yuehua Zhou, Xin Gao, Liucun Gao, Xifan Mei, Meiyan Sun, Zhuomei Zhang, Haifeng Song

**Affiliations:** ^1^Department of Biology, Liaoning Medical University, Jinzhou 121001, China; ^2^Institute of Psychology, Chinese Academy of Sciences, Beijing 100101, China; ^3^Department of Pharmacology and Toxicology, Beijing Institute of Radiation Medicine, Beijing 100850, China; ^4^Institute of Osteosarcoma, Tangdu Hospital, The Fourth Military Medical University, Xi'an 710038, China; ^5^Educational Technologies and Simulation Training Centre, Naval University of Engineering Tianjin Campus, Tianjin 300450, China; ^6^Shanghai Junshi Biosciences Inc., Shanghai 201203, China; ^7^Central Research Laboratory, Jilin University Bethune Second Hospital, Changchun 130041, China; ^8^Department of Gynecology and Obstetrics, General Hospital of Chinese Armed Police, Beijing 100039, China

## Abstract

Arginine is a semiessential amino acid required for the growth of melanoma and hepatocellular carcinoma, and the enzymatic removal of arginine by pegylated arginine deiminase (ADI) or arginase is being tested clinically. Here, we report a genetically engineered arginase FC fusion protein exhibiting a prolonged half-life and enhanced efficacy. The use of this enzyme to treat different tumor lines both inhibited cell proliferation and impaired cellular migration *in vitro* and *in vivo*. Our data reinforce the hypothesis that nutritional depletion is a key strategy for cancer treatment.

## 1. Introduction

Hepatocellular carcinoma (HCC) is the third most common cause of cancer death in the world, and the majority of patients with HCC are diagnosed at a late stage. Together with the lack of proven effective chemotherapy, the 5-year survival rate of HCC patients is only 7% worldwide [1]. 

Amino acids serve as regulatory molecules that modulate numerous cellular functions and provide the substrates for protein synthesis. Therefore, amino acid deprivation is an effective treatment strategy for some cancers. Asparagine depletion by L-asparaginase is one of the best known examples, and it is widely used in clinical oncology for the treatment of acute leukemia and certain lymphomas [2]. Seventy years ago, arginine, a semiessential amino acid, was demonstrated to control tumor growth in mice [3]. For normal cells, arginine is a nonessential amino acid. However, a number of different tumor types, including melanoma and HCCs, are unable to synthesize arginine and, as a result, are sensitive to treatments using arginine-degrading enzymes [4–6]. Indeed, pegylated forms of these enzymes have advanced into phase I and II clinical trials [7, 8]. 

Arginine deiminase (ADI) is a potent arginine-degrading enzyme from mycoplasma. Despite such concerns as immunogenic and toxic effects due to released ammonia [9], ADI was highly effective in the treatment of HCC and malignant melanoma. Arginase is another well-studied arginine-depriving enzyme acting in the urea cycle to convert arginine to ornithine and urea. Although arginine depletion by arginase leads to instant cell death in a variety of tumor cells [10], due to the short circulatory half-life of arginase, it was not pursued as a drug candidate [11] until 2007 when Leung's group demonstrated that pegylation circumvented this problem and led to satisfactory* in vivo* antitumor results in animals [12]. 

As a popular and efficient way to improve the biopharmaceutical properties of bioactive proteins and peptides, pegylation has been widely used to ameliorate the stability, solubility, and immunological properties of bioactive compounds. However, precautions must be taken when using this method. Firstly, pegylation produces a population of drug conjugates. Secondly, these conjugates can accumulate in the liver, which ultimately leads to macromolecular syndrome. As an alternative method, biomolecules can be linked to the human Fc region of human immunoglobulin (IgG1), with demonstrated unique advantages. Numerous effector molecules, such as soluble cytokine receptors, retain their respective biological activities after coupling to the Fc domain of IgG1. The binding of Fc to FcRn receptors, which serves an important function in IgG homeostasis, results in increased half-lives of the fusion constructs. 

Here, recombinant human arginase in the form of an Fc fusion protein (rhArg-Fc) was constructed and tested *in vitro* and* in vivo* for its antitumor activity. The results indicated that rhArg-Fc effectively inhibited the cellular growth of different tumors in culture and reduced tumor size in mice.

## 2. Material and Methods

### 2.1. HCC Xenografts in Nude Mice

Four-week-old athymic immunodeficient nude mice were obtained from Beijing Laboratory Animal Research Center. Approximately, 10^6^ Huh7 cells were removed from tissue culture plates using trypsin and injected *s.c.* into the back of the mice. The treatments were initiated after the size of each tumor reached approximately 5 mm in diameter. Six animals were used for each treatment group. The size of the tumors was monitored every 5 days for 50 days.

### 2.2. Cell Viability Determination Using MTT Assays

Cells were seeded in 24-well plates at a density of 2.5 × 10^4^ cells/well in 1 mL of culture medium and incubated for 24 h. The culture medium was replaced by a medium containing rhArg-Fc at different concentrations. After 3 days, the cellular growth and viability were measured using a tetrazolium salt assay [13]. The cells were incubated for 4 h at 37°C with tetrazolium salt (3-(4,5-dimethylthiazol-2-yl)-2,5-diphenyltetrazolium bromide), with the metabolically active cells reducing the dye to formazan. Dark-blue formazan crystals were dissolved in 1-propanol, and the absorbance was measured at 570 nm [9]. For the amino acid rescue experiment, the measurements were obtained after incubation with rhArg-Fc in RPMI 1640 medium supplemented with 10% FCS. The medium was removed by vigorous washing, and the cultures were maintained in arginine-free RPMI 1640 medium supplemented with 1mM L-Arg or L-Glu and incubated for another 3 days.

### 2.3. Amino Acid Analysis Using HPLC

Amino acid analyses were performed using an Agilent HPLC, as described in [13]. Briefly, the amino acids in the cell culture medium were extracted by mixing the medium with 75% methanol and precipitating the mixture at 4°C for 2 h. The samples were collected by centrifugation at 13,000 g for 30 min and measured using a cation exchange column and a fluorescence detector.

### 2.4. Pharmacokinetic Analysis of rhArg-Fc

Three CD1 mice were injected *i.v.* with 100 *μ*g/mouse and bled at 1, 2, 4, 6, 24, 72, and 144 h after injection. The levels of rhArg-Fc were measured with an ELISA assay using an antiarginase polyclonal antibody (Thermo Scientific) as the capturer. An antibody against the human Fc region (Jackson Immunology Research) was chosen as the reporter. 

### 2.5. *In Vitro* Scratch Assay

Human endothelial cells from umbilical cord vein were purchased from Allcells Inc. A confluent monolayer of synchronized HUVECS was scraped with a razor blade as described in [14]. After wounding, the cells were washed with PBS and incubated in fresh medium with 0.5 IU/mL rhArg-Fc or not 24 hours after the lesions were made; the coverslips were washed with ice-cold PBS and fixed in 4% formaldehyde.

### 2.6. *In Vitro* Cell Microvessel Formation Assay

Confluent HUVE cell monolayers were treated with 0.5 IU/mL rhArg-Fc or not for 48 h before being harvested and plated onto Matrigel-coated 24-well cluster plates (4 × 10^4^ cells/well) using medium that had been pretreated with 0.5 IU/mL rhArg-Fc or not for 24 hr. Microvessel formation was observed using an inverted light microscope at 40x.

### 2.7. Cell Cycle Analysis

Huh 7 cells were treated with rhArg-Fc at different concentrations for two days. Cells were washed with PBS, containing 1% BSA. Then, 3 mL of cold absolute ethanol was added, incubated at 4°C for 1 hr., washed, and provided with 1 mL of a 50 *μ*g/mL of propidium iodide in sodium citrate, and 50 *μ*L of a 10 *μ*g/mL RNaseA solution (R&D Systems, Minneapolis, MS; 200 U/mL). Cells were incubated for 3 h at 4°C and assayed by flow cytometry.

### 2.8. *In Vivo* Efficacy of rhArg-Fc on Nude Mice

10^6^ Huh7 cells were injected *s.c.* into the 4-week-old nude mice. The rhArg-Fc treatments were initiated after the size of the tumor reached approximately 5–10 mm in diameter. Six animals were used for each treatment group (cisplatin 0.5 mg/kg or rhArg-Fc 300 IU/mouse or a combination of both treatments). Mice were then treated twice a week for 2 weeks. The tumor size was monitored every 5 days for 50 days.

### 2.9. Statistical Analysis

All data were based on at least three independent experiments. For the *in vivo* data, statistical analysis was done with SPSS. The differences in tumor sizes were determined by two-tailed Student's *t*-test.

## 3. Results

### 3.1. rhArg-FC Specifically Degrades Arginine *In Vitro*

**Figure 1:**
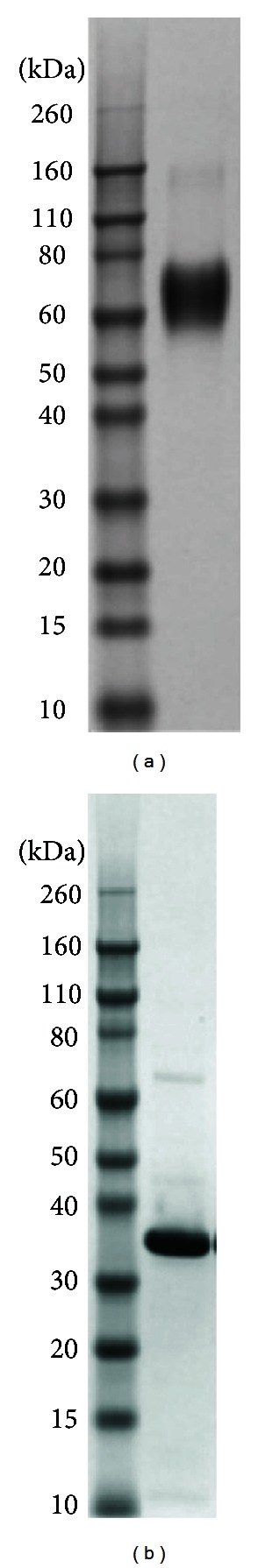
SDS-PAGE gel of rhArg-Fc and native arginase. (a) rhArg-Fc and (b) native arginase were purified to homogeneity, separated on an SDS-PAGE gel, and stained with Coomassie Blue; 2 *μ*g of protein was loaded in each lane.

**Figure 2:**
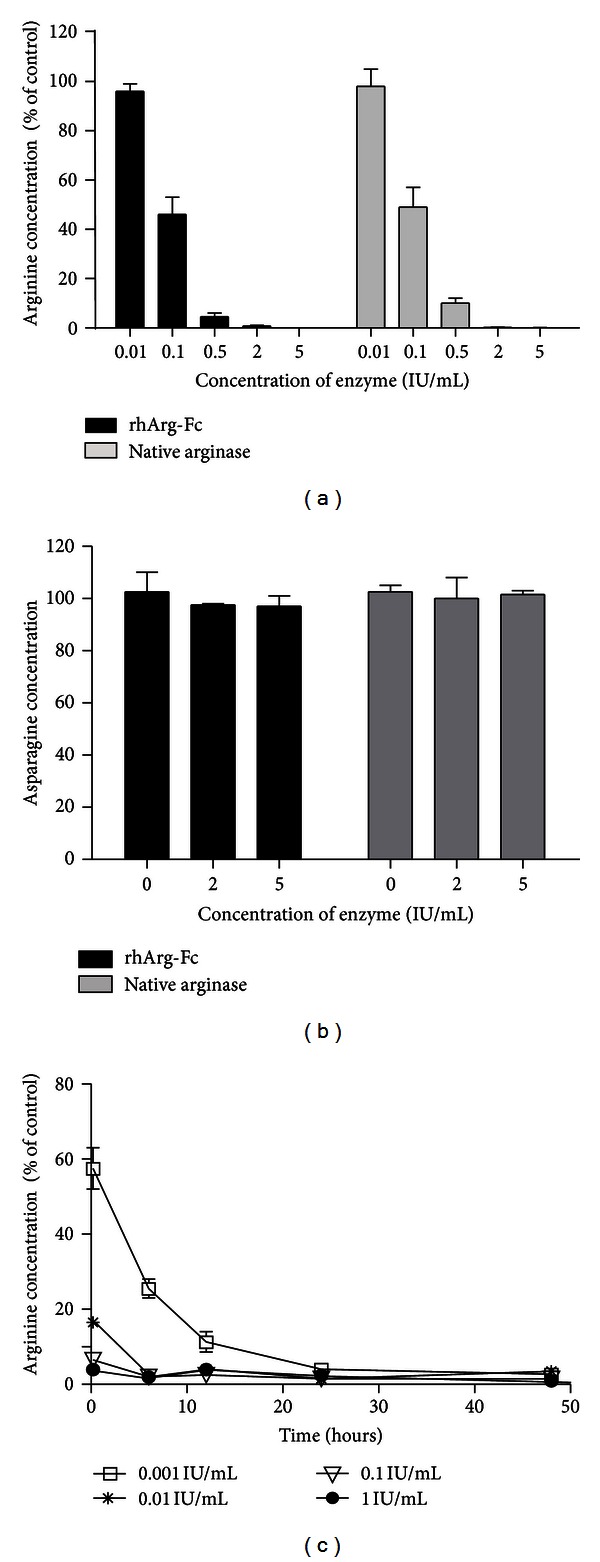
Enzymatic activity of rhArg-Fc. (a) and (b) native arginase or rhArg-Fc at the indicated concentration (nM) were incubated with 100 *μ*M arginine (a) or asparagine (b) in PBS for 2 h at 37°C. The amino acid concentration was determined by HPLC, as described in Section 2, and normalized to the sample without drug treatment. For the time-course experiment of the effect of rhArg-Fc on arginine depletion, rhArg-Fc at different concentrations was added to RPMI medium supplemented with 100 *μ*M of arginine. Each mixture with rhArg-Fc was divided into 5 groups and incubated at 37°C. The amino acids were extracted at the indicated time points, and the collected samples were quantified and normalized to the amount of the sample without rhArg-Fc treatment.

The rhArg-Fc fusion was created by fusing cDNAs encoding human arginase containing a hinge region and the CH_2_ and CH_3_ constant regions of the human IgG1 heavy chain. A vector directing the mammalian expression of rhArg-Fc was introduced into CHO cells to produce the secreted molecular protein. Taking advantage of the presence of the human IgG1 Fc region, we purified the fusion protein from the cell culture supernatant using a protein A column and eluted the column with acetate buffer (pH 3.8). We next examined the subunit structure of rhArg-Fc after SDS-polyacrylamide gel electrophoresis; the results showed that the subunit size is approximately 65 kd (Figure 1(a)), whereas that of the native form is 35 kd (Figure 1(b)). To test the ability of rhArg-Fc to catalyze arginine, different concentrations of rhArg-Fc or its native form were incubated at 37°C with serum-free RPMI medium supplemented with 100 *μ*M arginine; the medium was subjected to amino acid analysis after 2 h. At all the tested concentrations, rhArg-Fc was as potent as the recombinant human arginase, and a marked decrease in the arginine level was observed even in the low-dose treatment. Thus, the enzymatic activity of arginase is well preserved in its Fc-fusion form (Figure 2(a)). Furthermore, as rhArg-Fc did not decrease the level of asparagine, which was used as the negative control (Figure 2(b)), rhArg-Fc specifically uses arginine as a substrate. A similar result was reported by Cheng et al. [12], demonstrating that pegylated human arginase only degraded arginine and did not degrade the other tested amino acids.

To characterize rhArg-Fc further, we performed a pulse-chase experiment (Figure 2(c)) using RPMI medium supplemented with 100 *μ*M arginine. Ten minutes after the addition of 0.1 IU/mL or 1 IU/mL rhArg-Fc to the culture medium, the arginine concentration was reduced to below 5% of its original concentration. For all the tested rhArg-Fc dosages, the minimum value was reached within 24 h after the addition of the fusion.

### 3.2. Effect of rhArg-Fc on Human Tumor Cell Lines

**Figure 3:**
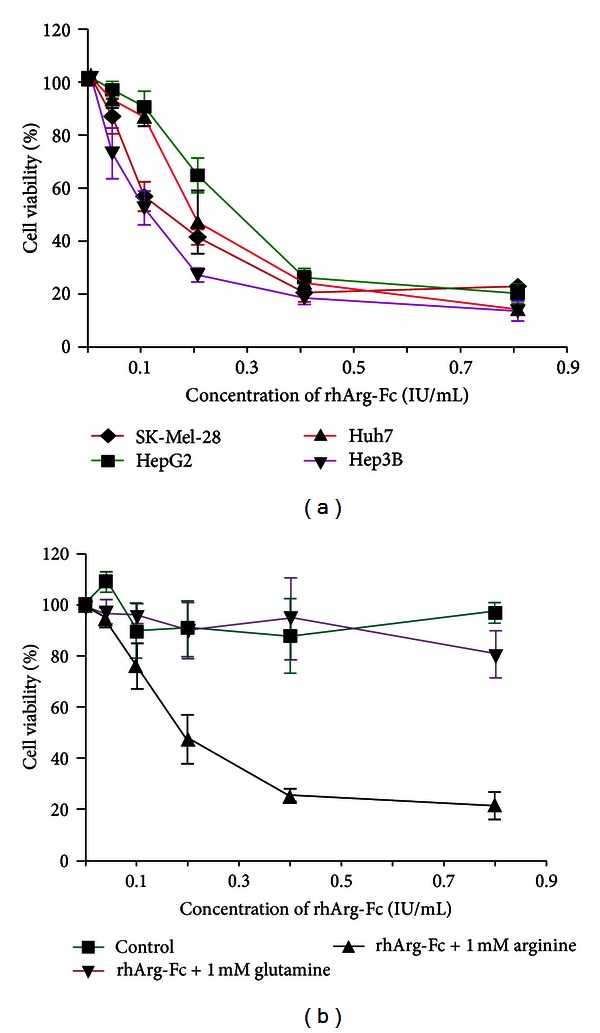
rhArg-Fc inhibits tumor cell growth. (a) Three HCC cell lines (HepG2, Hep3B, and Huh7) and 1 melanoma cell line (SK-Mel-3) were incubated with rhArg-Fc at different concentrations for 3 days. Cell viability was determined as described previously [9]. (b) Three days after rhArg-Fc treatment, the cells were washed extensively with PBS and incubated further in fresh medium containing exogenous 1 mM L-arginine or L-glutamine for an additional 3 days. The number of cells was determined as described in Section 2.

We then examined the cellular effect of rhArg-Fc in different cultured tumor cells. A dose-dependent inhibition of rhArg-Fc was observed in all of the tested cell lines, including 3 HCC cell lines (HepG2, Hep3B and Huh7) and 1 melanoma cell line (SK-Mel-3) incubated with rhArg-Fc at multiple concentrations for 3 days. All of the tested human tumor cell lines were sensitive to the inhibition of both forms of rhArgs (Figure 3(a)). The potency of rhArg-Fc had an IC_50_ ranging from 0.08 to 0.21 IU/mL, which is essentially equivalent to the value reported for its native form [12]. This result further suggests that the Fc fusion does not adversely affect the enzymatic function of arginase.

In a separate assay, three days after rhArg-Fc treatment at the concentration of 0.2 IU/mL, rhArg-Fc was removed by intensive PBS wash. The addition of exogenous 1 mM L-arginine, but not L-glutamine, almost fully resumed cell growth (Figure 3(b)). This further proved that rhArg-Fc inhibit cell proliferation through depletion of arginine but not the other amino acids.

### 3.3. Arginine Deprivation by rhArg-Fc Leads to Cell Cycle Arrest

**Figure 4:**
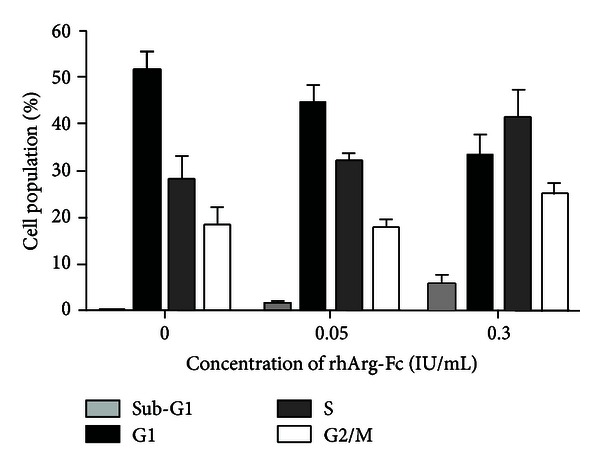
rhArg-Fc induces tumor cell arresting at S-phase. Huh7 cells were incubated with rhArg-Fc at different concentration for 2 days. Cell DNA content were analyzed by flow cytometry as described in the Materials and Methods. rhArg-Fc treatment induced a dose-dependent increase in the proportion of cell in S-phase while causing a decrease in the G1 phase compare to control.

To further investigate the effect of rhArg-Fc on cell growth in more detail, we analyzed the effects of rhArg-Fc on the cell cycle distribution of Huh7. This was monitored by flow cytometry analysis after staining the cell DNA content with propidium iodide. In comparison with untreated controlled cells, 2-day treatment of rhArg-Fc at the concentration of 0.1 IU/mL resulted in an apparent increase of S-G2 phase cells. In addition, cells respond to rhArg-Fc in a dose-dependent manner as there are more S-phase arrested cells in the cells treated with 0.5 IU/mL of dosage. The S-phase fraction increased from 28% in controlled cells to 43% in 0.5 IU/mL treated cells (Figure 4).

### 3.4. rhArg-Fc Treatment Inhibits Angiogenesis

**Figure 5:**
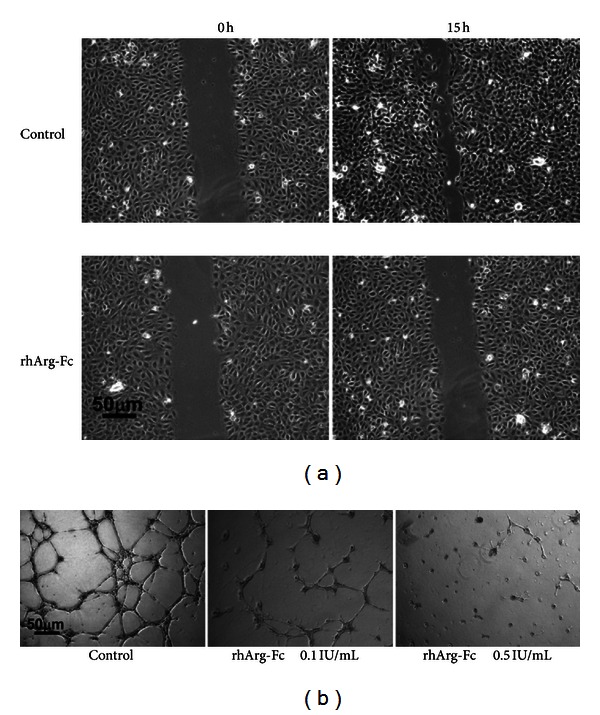
rhArg-Fc inhibits endothelial cell growth and migration. (a) Confluent HUVEC cells were wounded with a tip, washed and replaced with medium in the presence or absence of 0.5 IU/mL rhArg-Fc; images at 0 and 15 h were captured. (b) Confluent HUVEC cells were suspended in Matrigel in the presence or absence of rhArg-Fc; images were captured at 18 h.

The depletion of arginine by ADI was recently reported to inhibit endothelial cell growth and migration; this antiangiogenic activity might be due to the suppression of nitric oxide (NO) generation [15, 16]. To investigate the effect of rhArg-Fc function on angiogenesis, we first tested its effect on endothelial cell migration because endothelial cell mobility is an important factor in angiogenesis. Twenty hours after confluent HUVEC cells were wounded using a pipette tip, the wound was closed in the control cells; however, the healing process was significantly delayed in the rhArg-Fc-treated HUVEC cells (Figure 5(a)).

To further characterize the effect of rhArg-Fc on endothelial cell differentiation, we used a Matrigel assay to examine the effect of the fusion on microvessel formation, another key step in angiogenesis. Eighteen hours after treatment, the untreated endothelial cells differentiated into an elongated form and connected with each other to form networks. In contrast, rhArg-Fc significantly inhibited the differentiation of HUVEC cells into capillary-shaped tubes and resulted in sporadic vessel connection in a dose-dependent manner (Figure 5(b)). 

### 3.5. Pharmacokinetics and Pharmacodynamics of rhArg-Fc

**Figure 6:**
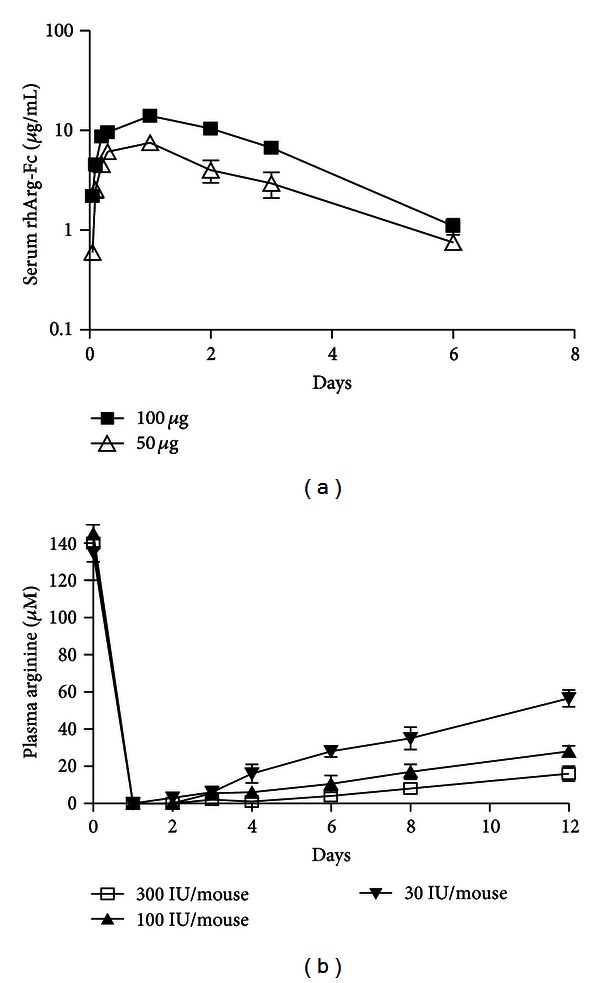
rhArg-Fc has an extended half-life *in vivo*. (a) Three CD1 mice were injected *i.v.* with 100 *μ*g or 50 *μ*g rhArg-Fc/mouse and bled at 1, 2, 4, 6, 24, 72 and 144 h after injection. The levels of rhArg-Fc were measured by ELISA. (b) The plasma levels of arginine were determined at 1, 2, 3, 4, 6, 8 and 12 days post-treatment with 30–300 IU/mouse of rhArg-Fc.

To determine the *in vivo* stability of rhArg-Fc, the pharmacokinetics of rhArg-Fc was measured following the *i.v.* injection of mice. Blood samples were collected on different days, and the serum rhArg-Fc levels were tested by ELISA. The half-life of rhArg-Fc was approximately 4 days for the different sample groups treated with various amounts of rhArg-Fc (Figure 6(a)).

To determine the pharmacodynamics of rhArg-Fc, the plasma levels of arginine in mice were tested at different time points after rhArg-Fc treatment. As shown in Figure 6(b), rhArg-Fc depleted arginine in a dose-dependent manner. For all the tested dosages, rhArg-Fc sharply reduced the circulating arginine levels to the undetectable levels on day 1. The highest dose of 300 IU per mouse maintained arginine at this undetectable level for more than 5 days (Figure 6(b)). 

### 3.6. *In Vivo* Effects of rhArg-Fc on Human HCCs Implanted into Mice

**Figure 7:**
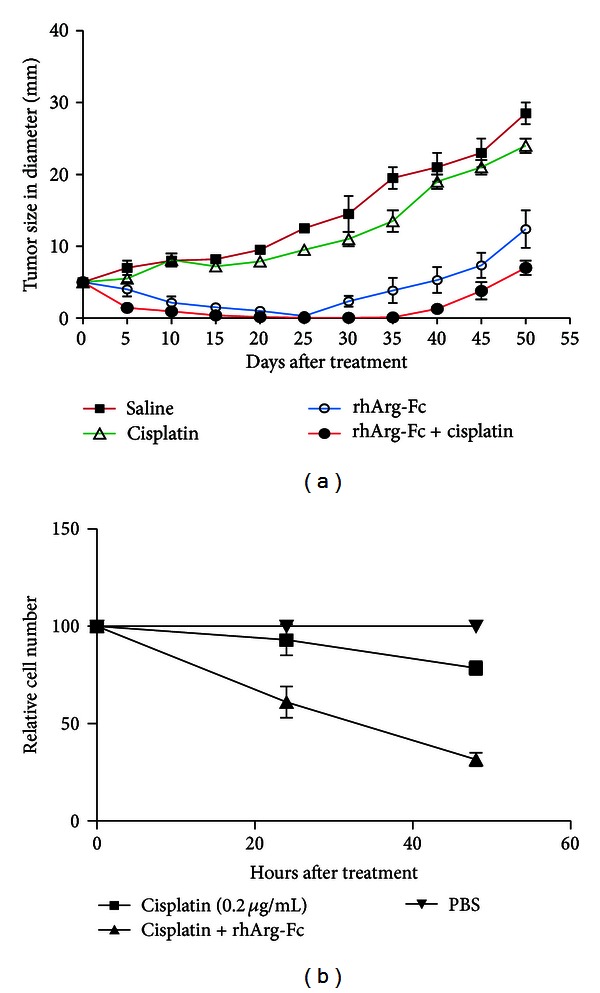
rhArg-Fc inhibits tumor growth* in vivo*. (a) 10^6^ Huh7 cells were injected *s.c.* into the 4-week-old nude mice. The rhArg-Fc treatments were initiated after the size of the tumor reached approximately 5–10 mm in diameter. Six animals were used for each treatment group (cisplatin 0.5 mg/kg or rhArg-Fc 300 IU/mouse or a combination of both treatments). The tumor size was monitored every 5 days for 50 days. (b) 10^5^ Huh7 were treated with 0.2 *μ*g/mL cisplatin in the presence or absence of 0.5 IU/mL rhArg-Fc. After 3 days, the number of cells was determined by MTT assay.

To determine the* in vivo* effects of rhArg-Fc, we examined its efficacy using a mouse xenograft model; Huh7 cells were implanted into nude mice, and the tumors were allowed to reach a size of 5 to 10 mm in diameter. Tumor growth was notably delayed in the mice that received 300 IU of rhArg-Fc twice a week, whereas progressive tumor growth was observed in saline-treated mice. Cisplatin has been widely used to treat hepatocellular carcinoma [17], and, when rhArg-Fc was used in combination with a low concentration of cisplatin (0.5 mg/kg), tumor growth was inhibited synergistically, with an augmentation in the tumor regression rate (Figure 7(a)). This synergy was further confirmed in cultured Huh-7 cells; in previous experiments, we determined that 0.2 *μ*g/mL cisplatin resulted in a 20% reduction in the cell growth of Huh-7 cells (data not shown). Indeed, the combined use of this concentration with rhArg-Fc at the same concentration led to a greater than 80% reduction in cell growth (Figure 7(b)). 

## 4. Discussion

Amino acid deprivation is an effective strategy to treat human cancers that are auxotrophic for nonessential amino acids, and L-asparaginase is a well-known growth inhibitory enzyme that is clinically used to treat acute lymphoblastic leukemia [18, 19]. Arginine is another semiessential amino acid required for rapidly proliferating cell. Accordingly, arginine is a potential target for the treatment of different types of cancer. A reduction in arginine concentrations via metabolic enzymes or by incubation in arginine-free medium led to the inhibition of tumor cell growth *in vitro* [20, 21]. Pegylated ADI has been demonstrated in cell culture and animals to be a potent enzyme for arginine deprivation; thus, it may be effective in killing malignant tumors. Although ADI is being tested in phase II clinical trials against HCC and malignant melanoma [22, 23], several disadvantages of this enzyme must be addressed. The first issue is with regard to the immunogenicity of this enzyme. ADI is an arginine-degrading enzyme from bacteria and is not an endogenous protein of the human body, and, although ADI is in a pegylated form, it is fairly stable *in vivo*. In phase II studies, an antibody against its pegylated form was reported to be detected 5 weeks after treatment [7, 22], which would compromise its long-term efficiency. Another drawback is that ADI converts arginine to citrulline, which can be recycled by arginine succinate sythetase (ASS) to rescue nutritional depletion, and many malignant cells are able to express ASS and are resistant to ADI treatment [23]. In contrast, ornithine fails to show this recovery because of the lack of ornithine transcarbamylase (OTC) in most hepatocellular cells [12]. Therefore, rhArg-FC produces ornithine, which cannot regenerate arginine by the urea cycle in the absence of OTC and leads to the permanent depletion of arginine. The action of arginase is swift in high capacity, which indicates that it can rapidly clear arginine from the cell culture medium. As reported by Philip et al. [24], 20 seconds is sufficient for 1 unit of arginase to complete the conversion of arginine to ornithine. Based on these considerations, arginase might be a better therapeutic enzyme in this aspect.

Although arginase has been tested in experiments for many years [24, 25], limited success was achieved *in vivo* until Leung's group designed a pegylated form [12]; these authors showed that pegylation increased the rhArg half-life, resulting in tumor growth inhibition *in vitro *and *in vivo*. However, there are limitations to the use of pegylation. The first limitation is with regard to the polydispersity of polymers, which leads to a population of drugs with different pharmacological properties. Secondly, as high molecular weight molecules, PEG polymers can accumulate in the liver and cause macromolecular syndrome [26]. An alternative to extending the half-life of the protein is to fuse the native protein to the Fc fragment of human IgG [27, 28]. This method both increases protein stability* in vivo *and retains its biological and therapeutic properties. In this study, we found that rhArg-Fc specifically and effectively metabolized arginine, which had a strong effect on malignant cells relative to normal cells (data not shown). No detectable arginine was observed one hour after incubation with rhArg-FC* in vitro*. Three days after treatment, rhArg-Fc inhibited cell growth in all of the 3 tested tumor cell types in a dose-dependent manner, with IC_50_ values ranging from 0.1 IU/mL to 0.3 IU/mL, which is comparable to its native form [12]. Our data in cultured hepatoma cells also demonstrated that, after the removal of rhArg-Fc from the culture, the remaining arginine allowed cell growth to resume. To confirm that this recovery was not due to the selection of resistant cells, we readded rhArg-Fc to the medium 3 days after recovery; as expected, the majority of cell growth was arrested (data not shown). Therefore, when the HCC cells were reconstituted in an arginine-rich environment, most of the cells that were arrested by arginine deprivation for 3 days were able to recover. This finding suggests that a cytostatic effect is at least partially responsible for the inhibition of cell growth, which is consistent with previous observations [8].

We further analyzed cell cycle and found that rhArg-FC significantly increased hepatoma cells in S-phase. This S-phase arrest may also result in the slightly elevated apoptosis represented as the sub-G1 cells shown in the FACS assay. Therefore, rhArg-Fc exerts its antitumor function though cytostatic (cell cycle arrest) and cytotoxic effects. Some other well-known antitumor agents such as cisplatin and irofulven also induce S-phase arrest to inhibit tumor proliferation [29]. The mechanisms under this cell cycle arrest are currently under investigation in our lab. One explanation may be the increased expression of cyclin A protein as previously reported [8]. We also found that after treatment, transcription levels of p27 and p21, two of the key cyclin kinase inhibitors, are lower than the control group as examined by real-time PCR.

We found that rhArg-FC inhibits cell growth, cell migration, and capillary vessel formation in cultured HUVE cells. This inhibition of angiogenesis might be attributable to several reasons. Arginine is an essential amino acid for protein synthesis and polyamine formation, and depletion of arginine by arginase disrupts the nutrient supply for endothelial cell growth because polyamines are indispensable for endothelial cell proliferation [30]. In addition, arginine is required for NO synthesis because the balance between NO and polyamine synthesis is regulated by the extracellular arginine level. Several groups have demonstrated an association between the level of NO and endothelial cell growth [31]. Therefore, the regulation of arginine levels and its relationship to polyamine and NO production has been suggested to play an important role in tumor angiogenesis [32].

Although the majority of HCC cells were killed 3 days after the rhArg-Fc treatment in the *in vitro* cell culture, we found residual cells that survived during this period, and this group of cells was viable even when a high concentration of rhArg-Fc was used to deplete arginine from the medium. When HCC cells are maintained above the critical rhArg-Fc level, it is plausible that all of the arginine-sensitive clones are replaced by cells that are relatively resistant to arginine deprivation. This result is in agreement with our *in vivo* animal data and the observations of other groups [5, 12, 26]. The removal of arginine by rhArg-Fc in nude athymic mice with subcutaneous HCC xenografts significantly reduced the tumor size in the first 12–20 days, demonstrating the rapid inhibition of sensitive clones. However, 20 days after treatment, the xenografted tumors became enlarged, suggesting the existence of resistant clones. As previously mentioned, ornithine can be converted back into arginine through the sequential actions of OTC, ASS, and argininosuccinate lyase (ASL). Although most HCC cell lines are reported to be deficient in either OTC or ASS expression [12], we cannot rule out the possibility that a subfraction of HCC cells was able to express both OTC and ASS, which would result in arginine autotrophy.

Cisplatin is one of the most widely used drugs to treat various types of cancers. The antitumor activity of this drug is due to DNA crosslinking, interactions with cell surface nucleic acids, and the inhibition of methionine uptake into tumor cells [31, 33]. At a low concentration, cisplatin alone did not inhibit tumor growth; however, cisplatin in combination with rhArg-Fc synergistically interacted in cell culture to hinder HCC cell growth. It is possible that arginine deprivation sensitized the HCC cells to cisplatin, which killed the clones that were resistant to rhArg-FC. This possibility is relevant to the effect of a low concentration of rhArg-Fc used in combination with a regular dose of cisplatin.
